# Heterogeneity of treatment effect: the case for individualising oxygen therapy in critically ill patients

**DOI:** 10.1186/s13054-025-05254-5

**Published:** 2025-01-28

**Authors:** Daniel S. Martin, Michael P. W. Grocott

**Affiliations:** 1 Peninsula Medical School, University of Plymouth, John Bull Building, Plymouth, UK; 2 Perioperative and Critical Care Theme, NIHR Southampton Biomedical Research Centre, University Hospital Southampton/University of Southampton, Southampton, UK

**Keywords:** Oxygen, Hypoxaemia, Randomised control trials, Critical care

## Abstract

Oxygen therapy is ubiquitous in critical illness but oxygenation targets to guide therapy remain controversial despite several large randomised controlled trials (RCTs). Findings from RCTs evaluating different approaches to oxygen therapy in critical illness present a confused picture for several reasons. Differences in both oxygen target measures (e.g. oxygen saturation or partial pressure) and the numerical thresholds used to define lower and higher targets complicate comparisons between trials. The duration of and adherence to oxygenation targets is also variable with consequent substantial variation in both the dose and the dose separation. Finally, heterogeneity of treatment effects (HTE) may also be a significant factor. HTE is defined as non-random variation in the benefit or harm of a treatment, in which the variation is associated with or attributable to patient characteristics. This narrative review aims to make the case that such heterogeneity is likely in relation to oxygen therapy for critically ill patients and that this has significant implications for the design and interpretation of trials of oxygen therapy in this context. HTE for oxygen therapy amongst critically ill patients may explain the contrasting results from different clinical trials of oxygen therapy. Individualised oxygen therapy may overcome this challenge, and future studies should incorporate ways to evaluate this approach.

**Graphical Abstract:**

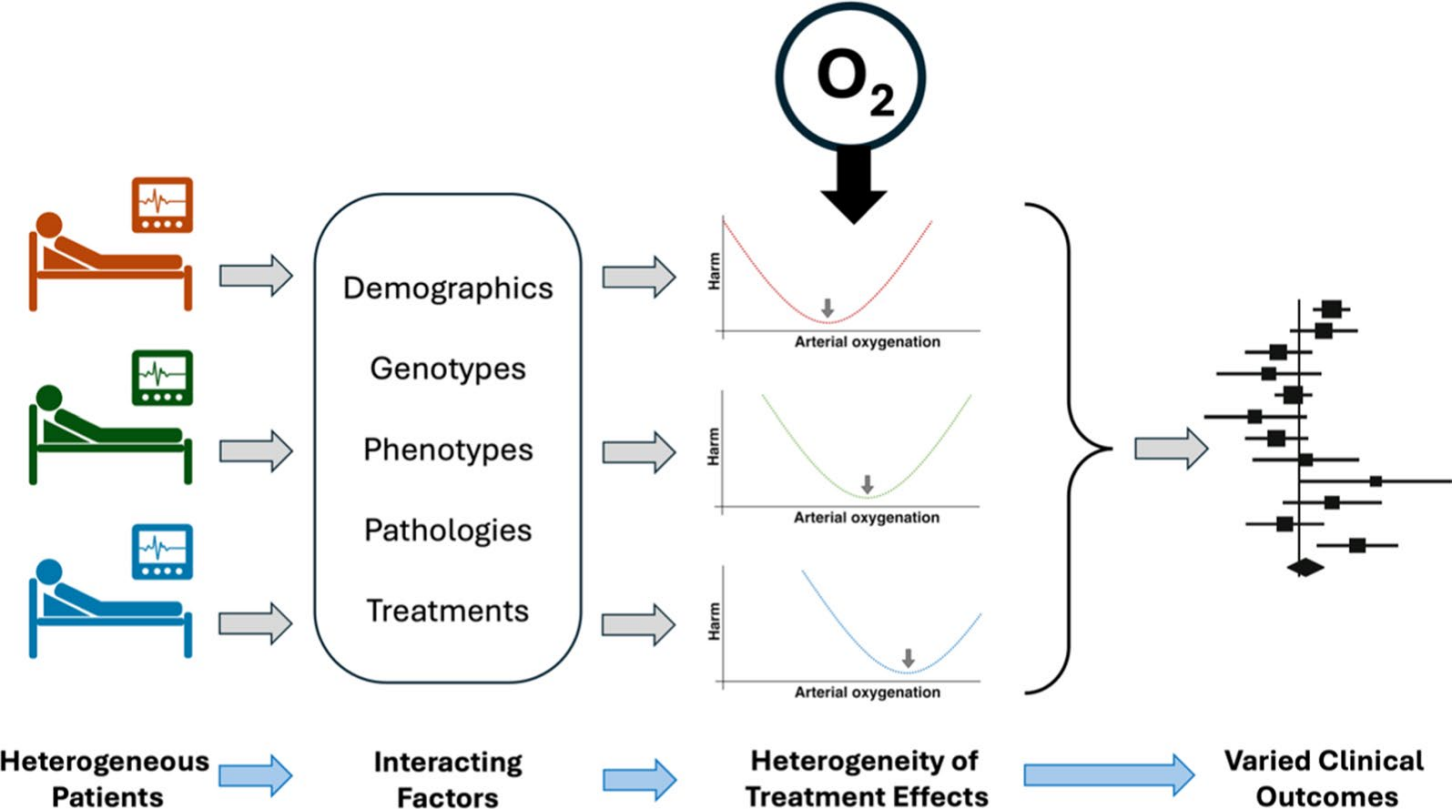

## Introduction

**Fig. 1:**
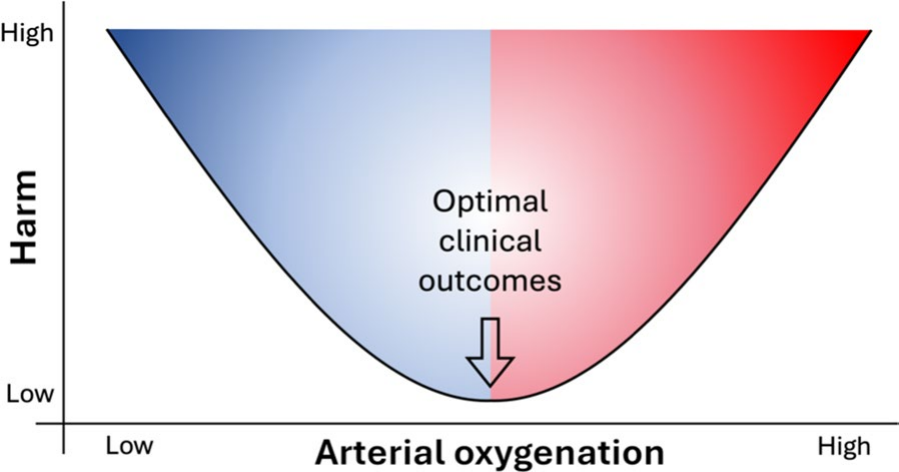
The proposed U-shaped relationship between arterial oxygenation and harm in an individual critically ill patient. Adapted from Martin et al. [4]

Oxygen is considered an essential therapy for most critically ill patients on intensive care units (ICUs) and life-saving for some, especially those with hypoxaemic respiratory failure. Additional oxygen is administered to supplement the oxygen in inspired air when the latter is no longer sufficient to maintain normal, or near normal, arterial oxygen levels (oxygen saturation of haemoglobin [SaO_2_] or partial pressure of arterial oxygen [PaO_2_]). Traditionally, clinicians have aimed to avoid hypoxaemia, when possible, to minimise the risk of cellular hypoxia and the organ dysfunction and failure that may accompany this. In practice, this desire to avoid hypoxaemia resulted in liberal use of supplemental oxygen, under the assumption that hyperoxaemia was harmless [1]. However, outside of intensive care medicine, the potential harm caused by high fractional inspired oxygen concentrations (FIO_2_) is well established [2, 3]. Consequently, questions were raised about the safety of using liberal concentrations of oxygen in critically ill patients [4]. A number of retrospective database analyses demonstrated relationships between oxygenation and mortality [1, 5–8] that led to the concept of a ‘U-shaped’ relationship between arterial oxygenation and mortality (Fig. 1) [4]. What these studies could not consistently answer, however, was the precise dose–response relationship and thresholds above which harm would be more likely. Importantly, the methods used do not support causal inference about the relationship between oxygenation and mortality. Building on this concept, several randomised controlled trials (RCTs) have addressed the question of whether more or less oxygen should be administered to patients [9–20]. Arguably, these trials have failed to bring us closer to a clear answer to the question we are trying to address: ‘how much oxygen should I administer to the patient I am caring for’.

This narrative review of the literature aims to discuss why we still do not know how much oxygen we should administer to critically ill patients, specifically focusing on the idea that heterogeneity of treatment effects (HTE) for oxygen could be the primary explanation for this.

## Oxygen can be harmful

The risks related to severe hypoxaemia require little discussion and those associated with excessive oxygen administration have been extensively reviewed by others [21–23]. In the context of critically ill patients, it is important to differentiate the direct effects of high concentration oxygen on the lungs (frequently referred to as oxygen toxicity) from the systemic effects of hyperoxaemia. In healthy humans, detectable oxygen toxicity is rare below an FIO_2_ of 0.5 [24]. The mechanistic explanation for pulmonary oxygen toxicity centres on production of reactive oxygen species (superoxide ions [O_2_^.−^], hydrogen peroxide [H_2_O_2_] and hydroxyl radicals [·OH]), which induce a state of oxidative stress, leading to lipid peroxidation, protein carboxylation and deoxyribonucleic acid oxidation [25]. Systemic hyperoxaemia induces coronary artery vasoconstriction [26], perhaps explaining why supplementary oxygen is not associated with any clinically important benefits in normoxaemic patients with acute myocardial infarction [27, 28] and may in fact be harmful [29]. Furthermore, humans possess an innate ability to adapt to moderate sustained hypoxaemia, albeit with substantial inter-individual variability in the rate and extent of response, as demonstrated when humans ascend to high altitude [30–32] and yet they have physiological minimal defence against oxidative stress beyond a relatively limited innate antioxidant system.

For acutely unwell adults the evidence to support a harmful effect of hyperoxia and/or hyperoxaemia is difficult to tease out from the literature. Whilst one systematic review and meta-analysis pooling data from more than 16,000 patients enrolled in 25 studies found liberal oxygenation strategies to be associated with increased mortality [33], no such association was found in a more recent and larger analysis [34].

## What have we learned from randomised trials of oxygen therapy?

Over the last decade the results of several RCTs evaluating conservative oxygen therapy have been published (Table 1). A full systematic review of that literature is beyond the scope of this article and is available in previously published work [35–38]. Most RCTs to date have set out to evaluate the benefit of interventions to reduce oxygen administration, commonly referred to as conservative oxygen therapy. An arterial oxygenation target (either PaO_2_ or SpO_2_) was used in these trials to encourage down-titration of FIO_2_ in the conservative oxygenation groups. From these trials and systematic reviews, no overall signal of benefit or harm has been demonstrated for conservative oxygen therapy. The possible reasons for this include:

- No true signal of benefit or harm exists.
- Variation between trials in the definition of conservative oxygen therapy (i.e. the oxygenation target).
- Variation between trials in the administration of oxygen therapy to patients in the comparator (control) group.
- Insufficient differentiation between intervention and comparator group oxygenation targets (including overlapping) within trials.
- Failure to achieve set oxygenation targets.
- Failure to achieve separation of oxygenation indices between intervention and comparator groups.
- Variation between trials in the type of patients being recruited.
- The existence of HTE for oxygen.

**Table 1:** Summary of key randomised controlled trials evaluating oxygen therapy in critically ill adult patients

Author	Trial name/acronym	Trial dates	Multi- or single centre	Intervention	Comparator	Number of participants analysed	General participant characteristics	Differences between groups for primary outcome
Asfar [20]	HYPER2S	2012–2014	Multi	FIO_2_ 1.0 for 24 h	SpO_2_ 88–95%	434	Mechanical ventilation with septic shock	28 day mortality was 43% in the hyperoxia group versus 35% in the normoxia group (HR 1·27, 95% CI 0.94–1.72; P = 0.12)
Barrot [17]	LOCO_2_	2016–2018	Multi	PaO_2_ 7.3–9.3 kPa OR SpO_2_ 88–92%	PaO_2_ 12–14 kPa OR SpO_2_ ≥ 96%	201	Mechanical ventilation with ARDS	No difference in 28 day mortality
Gelissen [15]	/	2015–2018	Multi	PaO_2_ 8–12 kPa	PaO_2_ 14–18 kPa	400	Critically ill with SIRS	No difference in organ dysfunction
Girardis [19]	Oxygen-ICU	2010–2012	Single	PaO_2_ 9.3–13.3 kPa OR SpO_2_ 94–98%	PaO_2_ ≤ 20 kPa OR SpO_2_ 97–100%, with FIO_2_ ≥ 0.4	434	ICU admissions	ICU mortality 11.6% in conservative group versus 20.2% in the comparator group (RR 0.57, 95% CI, 0.37–0.90; P = 0.01)
Mackle [18]	ICU-ROX	2015–2018	Multi	SpO_2_ 91–96%	SpO_2_ ≥ 91%, with FIO_2_ ≥ 0.3	965	Mechanical ventilation	No difference in ventilator-free days
Nielsen [9]	HOT-COVID	2020–2023	Multi	PaO_2_ 8 kPa	PaO_2_ 12 kPa	697	Hypoxaemic respiratory failure and COVID-19	At 90 days the median number of days alive without life support was 80.0 days in the lower oxygenation group and 72.0 days in the higher oxygenation group (P = 0.009)
Schjørring [16]	HOT-ICU	2017–2020	Multi	PaO_2_ 8 kPa	PaO_2_ 12 kPa	2888	Hypoxaemic respiratory failure	No difference in 90 day mortality
Schmidt [14]	BOX	2017–2021	Multi	PaO_2_ 9–10 kPa	PaO_2_ 13–14 kPa	789	Post cardiac arrest	No difference in 90 day mortality or hospital discharge with severe disability or coma
Semler [12]	PILOT	2018–2021	Multi	SpO_2_ 88–92% *SpO_2_ 92–96%	SpO_2_ 96–100%	2541	Mechanical ventilation	No difference in ventilator-free days
van der Wal [11]	ICONIC	2018–2021	Multi	PaO_2_ 7.3–10.6 kPa OR SpO_2_ 91–94%	PaO_2_ 14.6–20 kPa OR SpO_2_ 96–100%	664	Mechanical ventilation	No difference in 28 day mortality
Yang [53]	/	2017–2017	Single	SpO_2_ 90–95%	SpO_2_ 96–100%	168	ICU admissions	No difference in 28 day mortality

*FIO*_*2*_ fractional inspired oxygen concentration, *SpO*_*2*_ peripheral oxygen saturation, *PaO*_*2*_ partial pressure of arterial oxygen, *HR* hazard ratio, *ARDS* acute respiratory distress syndrome, *SIRS* systemic inflammatory response, *RR* relative risk

*Three group trial: low, intermediate and high oxygenation

Only three trials have shown differences in their primary outcome measure between approaches to oxygenation in critically ill adults. The first was in a single centre trial conducted in 2010–2012 that allocated 480 participants on ICUs to conservative or conventional oxygenation groups [19]. Mortality was reported as 11.6% in the conservative and 20.2% in the conventional oxygenation groups (p = 0.01). The trial received considerable criticism over its design and the results are not in line with those reported by others. The second is the HYPERS2S trial in which 442 patients with septic shock were recruited between 2012 and 2014 and randomised to receive an FIO_2_ of 1.0 for 24 h (hyperoxia group) or have oxygen titrated to achieve an SpO_2_ of 88–95% (described as usual care) along with either hypertonic or 0.9% sodium chloride during resuscitation in a 2 × 2 factorial trial design [20]. This trial was stopped early for safety reasons with an excess of deaths in the hyperoxia group (not reaching statistical significance) along with a higher incidence of serious adverse events. The findings from this trial are compelling evidence that a very high FIO_2_ is likely to be harmful to critically ill patients. More recently, a trial recruiting 726 patients with COVID-19 and severe hypoxaemia from 2020 to 2023 reported that targeting a PaO_2_ of 8 kPa resulted in more days alive without life support in 90 days than targeting a PaO_2_ of 12 kPa [9]. It is therefore possible that in patients with COVID-19, there is benefit in adopting a conservative approach to oxygen therapy. Additionally, in a trial recruiting 2040 mechanically ventilated children (aged 38 weeks corrected gestational age to 15 years) from 2020 to 2022, targeting an SpO_2_ of 88–92% resulted in greater probability of a better outcome in terms of duration of organ support at 30 days or death when compared with an SpO_2_ of > 94% [10]. Thus, whilst most trials to date have not demonstrated a difference in outcome between ‘conservative’ and ‘liberal’ oxygen therapy, interesting signals are emerging. In terms of the U-shaped curve concept; apart from the HYPERS2S trial, most trial findings only really tell us about a very small section in the middle of this conceptual curve, suggesting that this area may be a little flatter than previously imagined [39].

Two ongoing trials yet to report their findings may make a significant contribution to this field on account of their planned sizes. The UK-ROX trial being conducted in the United Kingdom has enrolled 16,500 participants [40] and the global MEGA-ROX trial is aiming to enrol 40,000 participants [41]. A priori sub-group analysis plans may provide a meaningful understanding of the differential effect of conservative oxygen therapy in sub-populations of critically ill patients, in other words, an insight into the heterogeneity of treatment responses to oxygen therapy.

## Heterogeneity of critically ill patients

We have known for a long time that patients admitted to ICUs are extremely heterogeneous; they can present with any diagnosis known to us today, spanning the entirety of surgery, medicine and mental health [42]. Whilst distinct diseases require specific treatments, clinicians are often battling diagnostic uncertainty, complex pathophysiology, and the sometimes hard to reconcile syndromes that we have created in an attempt to overcome these challenges. This heterogeneity amongst critically ill patients has hampered our ability to significantly improve their clinical outcomes [43]. The disappointing progress to date is not for lack of researcher effort. It is now common to see major clinical trials evaluating therapies in critically ill patients to be published weekly. Yet in recent decades, very few have reported substantial improvements in clinically important outcomes. In 2019, a systematic review of RCTs of trials in which any intervention or monitoring system were evaluated in critically ill patients and reported mortality as a primary or secondary outcome was conducted [44]. A total of 212 trials were included of which 170 (80%) reported no difference in mortality, 27 (13%) a significant reduction in mortality, and 16 (7%) an increase in mortality (one study was reported in 2 groups). Of the 27 trials that showed a reduction in mortality, several (all of which were pharmacological interventions) could not be replicated in subsequent RCTs. This contrasts with the COVID-19 pandemic, where participants in RCTs had a unifying diagnosis, thus were likely to exhibit considerably less heterogeneity, and several pharmacological treatments demonstrated clinical benefit [45, 46].

## Heterogeneity of treatment effects

HTE is defined as non-random variation in the benefit or harm of a treatment, in which the variation is associated with or attributable to patient characteristics [47]. Here, we make the case that such heterogeneity is likely in relation to oxygen therapy in critically ill patients and that this has significant implications for the design and interpretation of trials of oxygen therapy in this context.

Patients admitted to ICU form a heterogeneous population, even when we categorise them into syndromes such as sepsis and acute respiratory distress syndrome (ARDS). In addition, individuals, even within a given sub-population, respond differently to identical therapies, an example of HTE. In other words, when a treatment is administered to a group of patients, some may benefit from it, others may be harmed by it, and some may experience no effect at all.

RCTs are designed to identify a difference in the average effect of an intervention in one trial group versus no intervention in another trial group. The assumption of homogeneity of response is an important element of randomised comparisons, whereby the aim of parallel group randomisation within studies is to compare two alternative approaches based on the assumption that each approach will have similar effects in all patients.

**Fig. 2:**
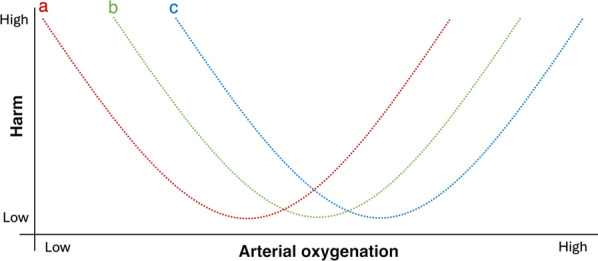
The potential relationship between arterial oxygenation and harm in a heterogeneous group of critically ill patients. **a**, **b** and **c** Individual responses to given levels of arterial oxygenation. In this example, least harm is associated with lower arterial oxygenation for individual **a** and higher arterial oxygenation for individual **c**, whilst **b** lies between the two. The potential for harm varies between individuals in such a way that altering oxygenation in one direction for the whole cohort may improve outcomes for some individuals but worsen them for others

Where this assumption is not valid, and there is substantial variation in patient response to interventions such that some patients may be benefiting from a particular intervention whilst others are harmed, then such randomised comparisons are likely to be misleading and futile [48]. Fundamentally, fixed numerical targets for PaO_2_ or SpO_2_ may not make sense in the face of substantial differences in individual physiology, in which case alternative targets for therapy may need to be used, based on an approach of endeavouring to identify the relevant target for each individual patient (Fig. 2). Moreover, amongst critically ill patients, there is wide variation in the risk of death and other adverse outcomes, which in turn means that there will be differences in the absolute benefit (or harm) any intervention might confer [49]. This can lead to scenarios where a trial reports an overall benefit of an intervention yet there is no benefit (or even harm) in a low-risk subset of the patients; or a trial reports no overall benefit of an intervention when considerable benefit actually exists in some high-risk patients [50]. Hence, the reported outcomes for a RCT are likely over-simplifying the true picture, and we risk discarding an intervention with considerable benefit, or accepting one that is harmful, to some participants.

The frequently made observation that trials of intensive care interventions commonly result in ‘no difference’ between groups may in part reflect these phenomena. It may be that treatments are not actually ineffective, but that we are not targeting them effectively to those who will benefit from them, whilst avoiding administering them to those who may not. The solution to this is effective individualisation of treatments, a goal that is easy to conceptualise but hard to achieve.

## Heterogeneity of treatment effects for oxygen

It is highly likely that human responses to supplemental oxygen and susceptibility to its side effects varies from person to person. At the other end of the oxygenation spectrum to hyperoxia, human responses to hypoxia are highly variable between individuals; for example, around 4% of those who successfully summit mount Everest (8848 m above sea level, where the equivalent oxygen concentration is approximately 7%) do so without the use of supplemental oxygen, whilst others are unable to reach its base camp at 5330 m. Similarly, susceptibility to high-altitude illnesses exhibits high inter-individual variability [51]. It is not unreasonable to posit that there may be much to learn from high altitude, where the dominant physiological challenge is hypobaric hypoxia, that may help explain phenotypes observed in critically ill patients nearer to sea level [52]. The observed inter-individual variation in responses to hypoxia are not explained by physical fitness or other physiological constructs and are likely to have their foundations in individual genetic and epigenetic differences [53, 54]. Whilst resilience to hypoxia is highly unlikely to be related to resilience to hyperoxia, the latter may also exhibit marked differences between individuals. Layered on top of our innate responses is the additional impact of an individual’s underlying pathophysiology, their responses to that pathophysiology, and potentially demographic factors such as age, sex and ethnicity. Therefore, the assumption that every patient will respond to hypoxia and supplemental oxygen therapy in a comparable way, leading to similar clinical outcomes, is unlikely to be valid. It is much more likely that a variety of different response profiles exist for different individuals (and even within the same individual at different times) (Fig. 2). This in turn represents a fundamental challenge to the internal validity of parallel group RCTs in this field to date.

Subgroup analysis of larger trials of oxygen therapy has provided some insight to the question of whether there is HTE for oxygen in critically ill adults (Table 2). One might expect conservative oxygen therapy to be advantageous post cardiac arrest as one of the key pathophysiological sequelae is hypoxic-ischaemic encephalopathy (HIE) following an ischaemia–reperfusion injury. This is a scenario where excessive oxygen in the circulation following the return of cardiac output may be detrimental to the brain [55]. In an individual-level patient data meta-analysis of RCTs where patients post cardiac arrest were randomised to receive either conservative or liberal oxygen therapy, conservative oxygen therapy was associated with a significant reduction in mortality at last follow-up compared to liberal oxygen therapy [56]. Yet, in an RCT recruiting 789 comatose patients post cardiac arrest, conservative (9 to 10 kPa) and liberal (13 to 14 kPa) oxygenation strategies resulted in a similar incidence of death or severe disability or coma [14]. It is important to note that the achieved separation in oxygenation indices between the two groups was considerably smaller than planned, a common finding in trials of conservative oxygen therapy [35]. In this trial, no average PaO_2_ values fell within the target range for the conservative group at timepoints within the first 48 h, which makes interpretation of the findings challenging. Whilst no differences were detected in primary or secondary outcomes in a subgroup of patients with sepsis all the point estimates favoured liberal oxygen therapy [57]. This perhaps makes sense given the pathophysiology of sepsis is classically described as involving tissue dysoxia [58]. Combining the data from the HOT-ICU [16] and HOT-COVID [9] trials in an individual patient data meta-analysis, the authors found HTE in 2 of 14 subgroups [59]. They detected lower mortality with conservative oxygen therapy for patients with cancer, and an increase in the number of days alive without life support for patients with COVID-19. Similar endeavours to compare, contrast and combine data from studies of oxygen therapy would benefit from alignment of approaches to data collection for all elements of trial conduct. The development of a core outcome set in this field merits consideration [60].

**Table 2:** Subgroup analysis findings from trials of conservative oxygen therapy in critically ill adults

Author and year	Primary trial	Subgroup population	Primary findings
Young [57]	ICU-ROX [18]	Sepsis (n = 251)	No difference in 90 day mortality between groups. However, point estimates for the treatment effect of conservative oxygen therapy raise the possibility of clinically important harm
Young [62]	ICU-ROX [18]	HIE (n = 166)	No difference in death or unfavourable neurological outcomes between groups at day 180
Young [63]	ICU-ROX [18]	Non-HIE acute brain pathology (n = 217)	No difference in 180 day mortality between groups
Klitgaard [64]	HOT-ICU [16]	Active haematological malignancy (n = 168)	No difference in 90 day mortality between groups
Crescioli [65]	HOT-ICU [16]	Post cardiac arrest (n = 355)	No difference in 90 day or 1 year mortality between the groups
Nielsen [66]	HOT-ICU [16]	COPD (n = 563)	No difference in 90 day mortality between the groups

*HIE* hypoxic–ischaemic encephalopathy, *COPD* chronic obstructive pulmonary disease

## Individualised oxygen therapy

Individualisation of therapy may involve both prediction of oxygen response phenotype to guide oxygen therapy targets and monitoring of responses to further refine individualisation during treatment. For example, demographic, clinical, genetic and epigenetic data may provide useful predictors of likely response. Monitoring of acute physiology during oxygen therapy (e.g. microcirculatory flow, perfusion, metabolic markers) may further refine such targets as the response to therapy becomes clear.

Recently, two trials of conservative oxygen therapy were combined into an analysis to determine whether an individual patient’s characteristics modified the effect of lower of higher oxygenation targets on mortality [61]. Using 28 day mortality as the primary outcome, the investigators developed a machine learning model to predict the effect of treatment with a lower vs higher SpO_2_ target from one large RCT [12] and externally validated the model using data from a second independent clinical trial [18]. They predicted that applying individualised SpO_2_ targets derived from this model to the derivation and validation trial participants could have reduced mortality by 6.4% [61]. As increasingly large and rich datasets become available from very large trials nearing completion in this area (e.g. the UK-ROX and Mega-ROX trials) it is likely that the performance of such models improves. Models like this may contribute to defining the oxygen targets for the next generation of trials evaluating individualised oxygen therapy. To realise such a vision, it is likely that both the sophistication of trial design along with the development and validation of oxygen response phenotypes and biomarkers (both biochemical and physiological) will need to be achieved.

## Conclusions

HTE for oxygen amongst critically ill patients may explain the contrasting results from different clinical trials of oxygen therapy and overall null effect reported to date when data are combined. Individualised oxygen therapy may overcome this challenge and future studies evaluating oxygen therapy in critical ill patients should be designed to enable evaluation of such approaches.

## Data Availability

No datasets were generated or analysed during the current study.

## Abbreviations

ARDS: Acute respiratory distress syndrome
DNA: Deoxyribonucleic acid
FIO_2_: Fractional inspired oxygen concentrations
HTE: Heterogeneity of treatment effects
HIE: Hypoxic-ischaemic encephalopathy
ICU: Intensive care unit
PaO_2_: Partial pressure of oxygen
RCT: Randomised controlled trial
SaO_2_: Oxygen saturation of haemoglobin
SpO_2_: Peripheral oxygen saturation
